# Enhancing COVID-19 Screening Models With Epidemiological and Mobility Features: Machine-Learning Model Study

**DOI:** 10.2196/54956

**Published:** 2026-03-05

**Authors:** Hyunwoo Choo, Dohyung Lee, Soo-Yong Shin, Jiwoo Lee, Duhun Lee, Eonji Kim, Namsoo Oh, Christina Kim, Myeongchan Kim, Hyo Jung Kim

**Affiliations:** 1 Department of Digital Health, Samsung Advanced Institute for Health Sciences and Technology (SAIHST), Sungkyunkwan University, Seoul, Republic of Korea; 2 Mobile Doctor Co., Ltd, Seoul, Republic of Korea; 3 Department of Biomedical Software Engineering, The Catholic University of Korea, 43 Jibong-ro, Wonmi-gu, Bucheon-si, Gyeonggi-do, 14662, Republic of Korea, +82-10-3093-1790

**Keywords:** deep learning, machine learning, COVID-19, mass screening, mobility data, epidemiology

## Abstract

**Background:**

Despite the significant post–COVID-19 pandemic surge in research using symptom data and machine learning (ML) for patient screening, data on patient trajectories and epidemiological conditions, although crucial, have remained underused.

**Objective:**

This study aimed to enhance the performance of ML models for COVID-19 screening by incorporating mobility and epidemic information in addition to patient symptom data.

**Methods:**

Data, including daily self-reported symptoms, location information, and test results, were collected from 48,798 individuals using a smartphone app. These data were then combined with Our World in Data and national government epidemic information to train 5 ML-based screening models to classify patient infection status. The models were logistic regression, extreme gradient boosting, light gradient boosting machine, tabular data network, and Google AutoML.

**Results:**

The addition of mobility and epidemic data significantly improved the performance of all 5 models. The highest area under the receiver operating characteristic curve score increased from 0.8712 without mobility and epidemic data to 0.9104 with mobility and epidemic data. This highlights the considerable impact of external information on enhancing the performance of ML models.

**Conclusions:**

This study demonstrated the potential of using mobility and epidemic data, such as location information and epidemic data, in combination with patient symptom data to improve the accuracy of ML models for diagnosing COVID-19. Considering additional contextual information can enhance the ability to screen for COVID-19.

## Introduction

Since the onset of the COVID-19 pandemic, rapid and accurate diagnostic methods have become crucial for controlling the spread of the disease. Although polymerase chain reaction (PCR) testing remains the gold standard for diagnosis [1], its limitations with respect to the need for specialized equipment, facilities, and time have hindered its scalability during the early stages of the pandemic [2,3].

In COVID-19’s early stages, screening strategies mainly focused on travel history and contact with infected individuals [4], primarily due to limited PCR capacity, as exemplified by the Centers for Disease Control and Prevention initially targeting international travelers for monitoring [5]. However, as community transmission increased, these methods were insufficient, leading to a shift toward symptom-based testing strategies [6]. This transition is supported by the fact that symptoms serve as primary indicators prompting testing for a range of infectious diseases, including influenza [7,8], Zika [9,10], malaria [11], and Ebola [12]. Particularly in areas lacking laboratory support, symptom-based screening has been recommended as an effective approach, as seen in the case of malaria [11].

Some research has shown promise in identifying potential COVID-19 cases using self-reported data and machine learning (ML) following a symptom-based screening method. Zoabi et al [13] developed an ML model that predicted COVID-19 test results with high accuracy using only 8 binary features, including demographic information and the presence of initial clinical symptoms [12]. Similarly, the COVID symptom study app in Sweden, which analyzed daily symptom reports from participants, developed a symptom-based model that outperformed traditional case notification-based models in predicting hospital admissions during the first wave of the pandemic [14].

However, the overlap of COVID-19 symptoms with those of other respiratory illnesses, such as influenza, has posed significant challenges in accurately identifying cases based on symptoms alone [15-19]. In addition, the presence of asymptomatic individuals infected with COVID-19 further complicates the accurate identification process, making reliance solely on symptom-based methods more challenging [20].

On the other hand, research on mathematical models that predict infectious disease prevalence and understand transmission dynamics, incorporating surveillance data, offers vital insights into public health strategies while also emphasizing the significance of epidemiological factors [21-23]. These models often consider factors such as traveling population, mobility, and contact patterns to better understand disease spread and inform control measures. Choo et al [24] show that combining epidemiological data with individual health data can enhance screening model accuracy. Loo et al [25] show that using mobility data enables the identification and characterization of superspreaders. These findings suggest that integrating community-level data and transmission dynamics into individual screening strategies could potentially enhance the effectiveness of COVID-19 screening as well, bridging the gap between broad epidemiological insights and actionable, individual-level interventions.

Given the constraints of symptom-based screening for COVID-19 and the potential advantages of incorporating epidemiological factors and mobility data, a combined approach could enhance screening efficacy. Previous studies have demonstrated the effectiveness of mobile apps in gathering symptom-based survey data and diagnostic test results [12,13]. Building upon this, we developed the SHINE (Study of Health Information for Next Epidemic; AI/DX Convergence Business Group) app to focus on collecting data for testing our combined approach. The app gathers symptom data via a smartphone interface, with users reporting specific symptoms outlined in the methods section. In addition, the app’s design facilitates the integration of mobility data and local surveillance indices, enabling the creation of a more comprehensive screening tool that considers the nonspecific nature of COVID-19 symptoms and the impact of epidemiological factors on disease spread. The app interacts with patients who have undergone diagnostic tests by enabling them to submit screenshots or photos of their results, which are then validated by the app’s reviewers to ensure data accuracy. This validation process aims to address the limitations of existing patient-generated health data platforms, where diagnosis data are often self-reported and may lack robustness for training ML models. By integrating verified diagnostic test results, the SHINE app aims to furnish a more dependable dataset for training precise COVID-19 screening models.

We postulated that merging symptom-based profiling of COVID-19 with mobility data and local surveillance indices could yield a more precise and resilient screening tool, acknowledging the nonspecificity of COVID-19 symptoms. Thus, our study endeavors to assess the efficacy of this integrated approach in enhancing COVID-19 screening strategies.

## Methods

### Study Design

**Figure 1.**
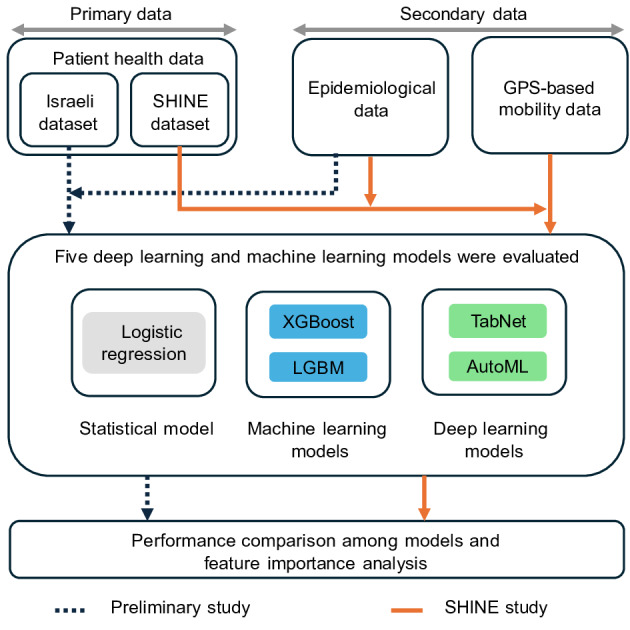
Overview of the study design. AutoML: automated machine learning; LGBM: light gradient boosting machine; SHINE: Study of Health Information for Next Epidemic; TabNet: tabular data network; XGBoost: extreme gradient boosting.

The main goal of this study is to demonstrate that deep learning models can better classify COVID-19 infections by incorporating additional data sources such as GPS-based mobility data and publicly available COVID-19 metrics alongside daily self-check symptom data, which serves as the primary input. In Figure 1, the overall study design is depicted. We used the SHINE dataset to train and evaluate 5 different ML models for COVID-19 screening. This study adheres to the CONSORT-AI (Consolidated Standards of Reporting Trials–Artificial Intelligence) reporting guideline.

Prior to our main analysis, we conducted a preliminary experiment using a public dataset from Israel [26], which demonstrated potential improvements in the predictive performance of ML models by incorporating readily available COVID-19 epidemiological data (Multimedia Appendix 1). This preliminary work supported our hypothesis that contextual information beyond patient symptoms could enhance screening accuracy.

For our main experiment, we leveraged the SHINE dataset, which contained rich contextual information, including mobility data obtained through GPS tracking. In this experiment, symptom data and demographic information were used as primary variables, and the analysis was extended by incorporating 3 levels of epidemiological data (global, national, and regional) along with mobility data as supplementary contextual variables. The main experiment evaluated the impact of these variables by adding their combinations to the base variables, allowing us to observe how additional contextual information affected the models’ predictive performance.

### Data Source

The SHINE dataset was collected through our proprietary platform called SHINE. As part of the platform, the SHINE app enabled daily self-check submissions from users, providing crucial demographic information such as age, sex, underlying health conditions, and mobility patterns obtained through smartphone location data [27]. Over approximately 3 years, we collaboratively developed and maintained the SHINE smartphone app with hospitals and private organizations to gather personal health data from users. Concurrently, an additional database was established by incorporating case statistics from various cities and districts provided by the central and local governments.

For contextual epidemiological information, we incorporated relevant data from Our World in Data, including global and national surveillance data [28].

#### SHINE Dataset

Self-check symptoms and authorized COVID-19 test results, along with basic information such as biological sex and age, were collected from October 2020 to March 2023 using the SHINE mobile app in South Korea. Some individuals submitted self-reported data without reporting their authorized COVID-19 test results, and vice versa. Notably, as the government’s standards for confirming positive COVID-19 cases varied over time, there was a mix of outcomes from different types of tests, such as PCR and rapid antigen tests. To enhance the reliability of the data, authorized government-issued documents were manually reviewed.

Data from users who previously submitted the results of authorized COVID-19 tests at least once were selected, regardless of whether the test results were positive or negative. Subsequently, the self-checked symptom data of the users were grouped into episodes based on their unique user identifiers. Each episode was then limited to a range of 2 weeks before and after the individual’s authorized COVID-19 test date.

During this process, we identified a subset of users who reported more than 2 authorized COVID-19 test results, along with their corresponding self-checked symptoms. Instead of excluding these users to prevent confounding owing to repeated testing at short intervals, we ensured that the episodes generated by the same individual were kept in the same group when splitting the data into training and testing sets.

#### Data Extraction and Episode Definition

We defined an “episode” as a 14-day window around each authorized COVID-19 test date. If a user had multiple tests on different dates (eg, 2 weeks apart), each test was treated as a separate episode. For each episode, we extracted the day of self-reported data that was closest to the test date. This meant that each episode ultimately yielded 1 single-day observation representing symptoms, mobility, and epidemiological data.

Some users underwent multiple COVID-19 tests, generating distinct episodes (single-day observations) per test. To prevent data leakage arising from individual-specific patterns (eg, demographics and behaviors) across episodes, all episodes from the same user were exclusively allocated to either the training or test set. This ensured model evaluation reflected generalization to unseen individuals rather than memorization of recurring user traits.

### Ethical Considerations

This study was approved by the Institutional Review Board of Sungkyunkwan University (IRB No: SKKU-2023-04-047 and SKKU 2022-11-014). Informed consent was waived because the entire dataset is anonymized, preventing researchers from accessing sensitive personal information and minimizing potential risks. No compensation was provided to participants since only deidentified secondary data were used.

## Results

### Mobility Data

The mobility data used in this study were obtained from GPS tracking data collected using the SHINE app. The raw GPS positions consisted of latitude and longitude coordinates along with timestamps. Our hypothesis was that increased movement might correlate with a higher risk of COVID-19 infection, so we extracted 3 different metrics to capture distinct aspects of patient mobility:

1. Number of GPS signals captured daily: this metric reflects the frequency of GPS signal capture, which correlates with the amount of time a patient is traveling.
2. Daily travel distance: calculated using the Haversine formula, this metric measures the distance traveled each day between recorded GPS points. The Haversine formula is appropriate for GPS data as it calculates the distance between 2 points on a sphere.
3. Dispersion of movement: this metric quantifies the extent of the area a patient visited each day. We assessed the spread of GPS coordinates by measuring longitudinal and latitudinal variance and then taking the root mean square sum of these variances.

These metrics were chosen to represent different dimensions of mobility: frequency of travel, distance traveled, and area covered. Together, they provide a comprehensive view of a patient’s mobility patterns and are well-suited to explore the relationship between mobility and COVID-19 risk.

To account for variations among users and days, the raw GPS data were reorganized into daily time series for each participant. Both daily travel distance and daily travel dispersion were then quantile-normalized. Further details, including data preprocessing steps, can be found in Multimedia Appendix 1 (location data preprocessing). COVID-19 epidemiological data COVID-19 epidemiological data encompass global-, national-, and regional-level information, offering insights into various variables such as daily confirmed cases, vaccination rates, and critical patient ratios. For global- and national-level data, we sourced information from Our World in Data, which aggregated data from esteemed institutions such as Johns Hopkins University and the World Health Organization. These data were used to formulate derived variables, including metrics such as the incidence of new cases, vaccination rates, and hospitalizations over a 6-month period, and capture the plausible emergence of herd immunity. Regional-level data, specifically at the province/city level, were obtained from data announced by the Korean health authorities. Data within the same category have undergone minimum-maximum normalization.

### Models and Training

#### Model Selection

Overall, 5 models widely used in the medical field, particularly in relevant studies, have been selected [29-31]. The aim is to showcase that our approach can enhance the performance of various models, regardless of their architectural nuances or inherent biases, as each model inherently carries its distinctive inductive bias. We chose 1 statistical method, 2 classical ML methods, and 2 deep learning methods. The following provides concise explanations of the selected models:

1. Logistic regression [32,33]: a statistical model that combines the weighted sum of variables with a sigmoid function, providing a straightforward understanding of the relationship between variables and outcomes.
2. Light gradient boosting machine [34] and extreme gradient boosting [35]: classical ML models widely used for structured table-form data. They leverage powerful algorithms for boosting decision trees, enabling accurate predictions.
3. Tabular data network (TabNet) [36]: a deep-learning model specifically designed for tabular data. TabNet uses a unique architecture called “attentive transformer” and uses unsupervised learning to enhance performance.
4. Google AutoML [37]: we incorporated Google’s AutoML, using the functionalities provided by Google Cloud’s Vertex AI. AutoML offers the unique capability to autonomously generate customized neural network architectures. The AutoML model was trained using the same training, validation, and test datasets as the other table-form learning models (Multimedia Appendix 1).

#### Training Setup

We collected a comprehensive dataset consisting of episodes related to COVID-19 cases, including both positive and negative episodes. To address any potential disparity in the number of positive and negative episodes, we combined the dataset and divided it for training and testing in an 8:2 ratio. To minimize confounding factors, episodes from the same individual were grouped.

To ensure a fair comparison and effective model training, we kept the test set fixed and randomly sampled the validation and training sets. For the models that did not require a validation set, 80% of the data were randomly selected from the training set. The entire experiment was repeated 5 times to account for inherent variability, and the averages and standard deviations of the results were calculated.

Some models used in this study do not inherently support time-series data, so we adopted a modified approach for data extraction. For each episode, we extracted data from the day nearest to the COVID-19 test date, creating a single observation per episode. This approach allowed us to capture relevant information within a limited timeframe while maintaining a consistent evaluation framework. For nontime-series models (logistic regression, light gradient boosting machine, and extreme gradient boosting), this single-day extraction was necessary, while time-series models (TabNet and Google AutoML) could naturally handle sequential data but were evaluated using the same extracted data points for fair comparison (Multimedia Appendix 1).

#### Performance Evaluation

To consistently evaluate the models’ performance, we used a comprehensive set of metrics, including the area under the receiver operating characteristic curve, area under the precision-recall curve, and *F*_1_-score.

#### Model Interpretability With Shapley Additive Explanations

We used Shapley additive explanations (SHAP) [38] to interpret model predictions. After training and evaluating all 5 algorithms, we selected the best-performing model—Google AutoML trained with the full (“All”) feature set—and calculated mean SHAP values for the entire test set. This provided global explanations of the model’s behavior, showing how symptoms, mobility, and epidemiological features contributed to predictions.

### SHINE Dataset Characteristics

A total of 48,798 unique users reported 3,571,243 daily self-checked symptoms and provided basic information, such as age and sex in the SHINE dataset, from October 2020 to March 2023. Over the same period, 17,298 unique users reported 21,773 authorized COVID-19 test results. A total of 15,351 patients reported at least 1 self-reported symptom and an authorized COVID-19 test result, irrespective of their test results. Overall, 5119 negative episodes (33.35%) and 10,232 positive episodes (66.65%) were recorded. Data characteristics are summarized in Table 1.

**Table 1.** Characteristics of the Study of Health Information for Next Epidemic dataset episodes used for model development.

Category		SHINE^a^ dataset (N=15,376)
Age (years), n (%)		
<60		14,427 (93.98)
≥60		924 (6.02)
Sex, n (%)		
Female		9928 (64.56)
Male		5423 (35.26)
Test indication, n (%)^b^		
Others		11,259 (73.34)
Contact^c^		3985 (25.96)
Abroad^d^		107 (0.70)
Test result, n (%)		
Negative		5119 (33.35)
Positive		10,232 (66.65)

a SHINE: Study of Health Information for Next Epidemic.

b Reasons prompting COVID-19 testing.

c Close contact with a confirmed COVID-19 case.

d Recent return from international travel.

The data covered a period of 2 weeks before and after the authorized COVID-19 test date, with most episodes lasting within 5 days. The SHINE app was predominantly used by the users to monitor their conditions after the test. We observed a sex imbalance in the dataset, with a significantly higher proportion of females than males (9928 female patients [64.56%] vs 5423 male patients [35.26%]). Comparison of the COVID-19 confirmation rate between sexes revealed no significant differences.

### Symptoms in the SHINE Dataset

The symptom distribution was not significantly different by age. All symptoms were significantly more prevalent in the positive group, suggesting robust differences between positive and negative episodes, as shown in Table 2.

**Table 2.** Comparison of symptom prevalence between the COVID-19–positive and -negative groups in the Study of Health Information for Next Epidemic Dataset. The Mann-Whitney test results indicate a significantly higher prevalence of all symptoms (*P*<.001) in the positive group.

Symptoms, n (%)	Positive group (n=13,977)	Negative group (n=6608)	PPV^a^
Cough	13,542 (96.88)	3652 (55.29)	0.7770
Sore throat	11,529 (82.47)	2161 (32.69)	0.8373
Headache	10,788 (77.21)	1986 (30.04)	0.8394
Sputum	8290 (59.29)	906 (13.72)	0.9033
Runny nose	4960 (35.51)	823 (12.46)	0.8551
Chills	1908 (13.66)	174 (2.63)	0.9172
Muscle pain	1872 (13.39)	302 (4.57)	0.8639
Fever	3913 (28.01)	302 (4.57)	0.1234
Shortness of breath	951 (6.81)	95 (1.44)	0.9034
Loss of taste	960 (6.87)	55 (0.83)	0.9392
Loss of smell	1000 (7.15)	58 (0.88)	0.9384
Diarrhea	930 (6.59)	107 (1.57)	0.8968

a PPV: positive predictive value.

**Figure 2.**
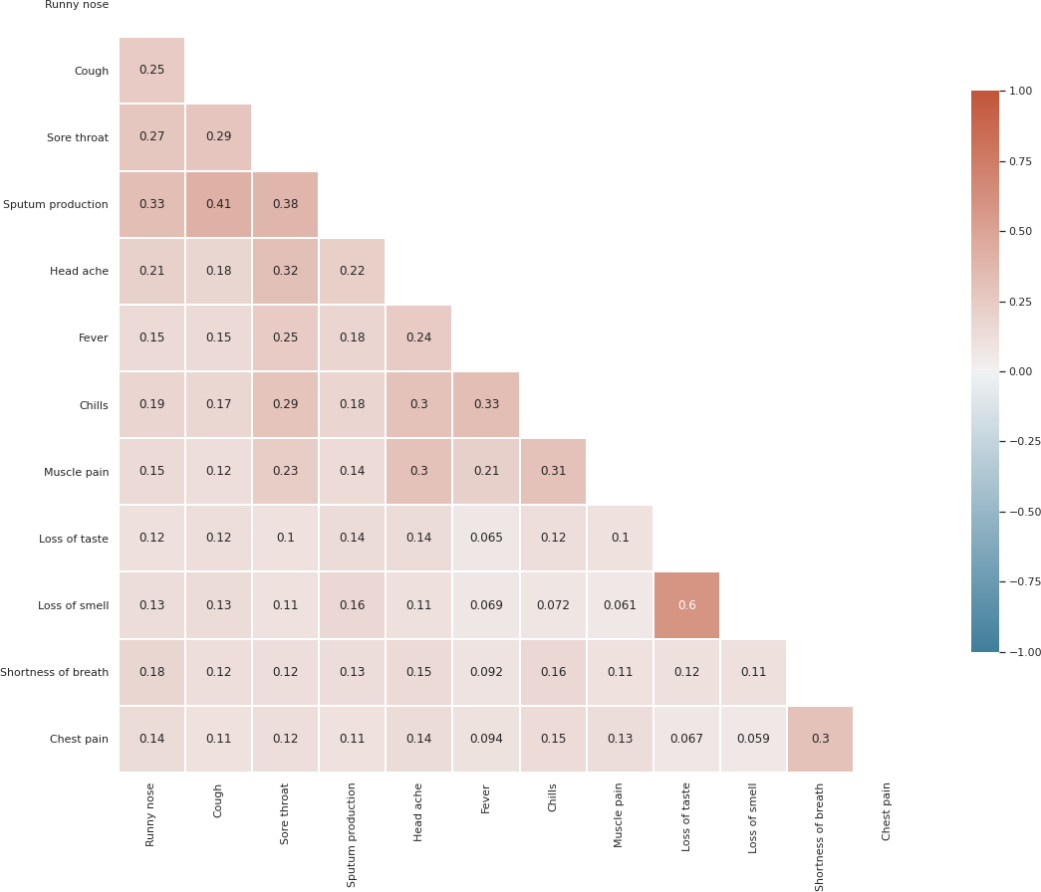
Symptom correlation in the Study of Health Information for Next Epidemic dataset. The Spearman correlation coefficients are displayed in the grid. There was a relatively strong correlation between loss of smell and loss of taste (0.6) and a moderately positive correlation between chills and fever (0.33). While some symptoms tend to co-occur more frequently, the overall pattern of symptom presentation is varied.

In addition, the symptoms were correlated, as shown in Figure 2. For instance, there was a relatively strong correlation between loss of smell and loss of taste (Spearman correlation coefficient: 0.6) and a moderately positive correlation between chills and fever (Spearman correlation coefficient: 0.33). In addition, there were relatively weak correlations among upper respiratory tract symptoms, including cough, sputum production, and sore throat.

**Figure 3.**
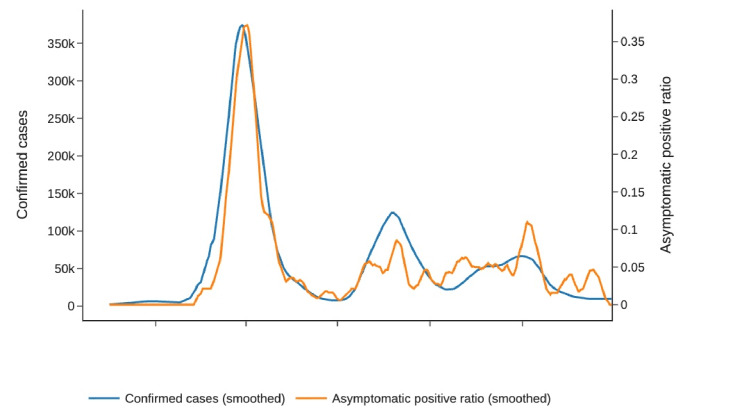
Comparison of 7-day moving average values for confirmed cases and asymptomatic positive ratio in the Study of Health Information for Next Epidemic dataset. The Spearman rank correlation coefficient between the 2 metrics is 0.7141 (*P*<.001), highlighting the persistent presence of asymptomatic cases throughout the pandemic period.

In Figure 3, a comparative analysis of the average confirmation rates in Korea over a span of 7 days showed a robust correlation between the asymptomatic confirmation rate and the overall confirmation rate (Spearman rank correlation coefficient=0.7141; *P*<.001). The asymptomatic confirmation rate was defined as the ratio of users reporting COVID-19 infections without exhibiting any symptoms to the total number of users who reported COVID-19 infections, irrespective of their symptom profiles.

### Impact of Mobility and Epidemiological Feature Addition on COVID-19 Prediction in the SHINE Dataset

Using the SHINE dataset, we conducted an experiment aimed to evaluate the improvement in performance achieved by selectively incorporating different groups of features into the baseline model, as shown in Table 3.

**Table 3.** Model performance in predicting COVID-19 with the integration of secondary features using the Study of Health Information for Next Epidemic dataset. Each row represents model performance after adding the indicated feature groups on top of the baseline.

Metrics (mean)		LR^a^	XGBoost^b^	LGBM^c^	TabNet^d^	AutoML^e^
Baseline^f^						
AUROC^g^		0.8646	0.8641	0.8643	0.8629	0.8712
AUPRC^h^		0.9286	0.9283	0.9286	0.9260	0.8686
*F*_1_-score		0.8566	0.8538	0.8511	0.8555	0.8051
+Mobility^i^						
AUROC		0.8732	0.8751	0.8797	0.8692	0.8926
AUPRC		0.9320	0.9311	0.9362	0.9293	0.8892
*F*_1_-score		0.8602	0.8565	0.8578	0.8531	0.8260
+Epidemiological^j^						
AUROC		0.9005	0.8978	0.9011	0.8956	0.9060
AUPRC		0.9443	0.9419	0.9450	0.9401	0.9040
*F*_1_-score		0.8761	0.8765	0.8766	0.6994	0.8349
All^k^						
AUROC		0.9008	0.8983	0.9046	0.8934	0.9104
AUPRC		0.9436	0.9413	0.9471	0.9378	0.9084
*F*_1_-score		0.8785	0.8745	0.8769	0.8730	0.8355

a LR: logistic regression.

b XGBoost: extreme gradient boosting.

c LGBM: light gradient boosting machine.

d TabNet: tabular network.

e AutoML: automated machine learning.

f Baseline: symptom + demographic variables only.

g AUROC: area under the receiver operating characteristic curve.

h AUPRC: area under the precision-recall curve.

i +Mobility: baseline plus 3 GPS-derived mobility metrics.

j +Epidemiological: baseline plus global·national·regional COVID-19 indices.

k All: baseline plus both mobility and epidemiological features.

Significant performance improvements were observed when COVID-19 epidemiological data were incorporated into the baseline model, with these improvements surpassing those achieved by integrating the mobility pattern data (mean difference in area under the receiver operating characteristic curve: 0.03 vs 0.01 for all models, Mann-Whitney test; *P*<.001). Notably, the improvement in model performance was greater with the inclusion of COVID-19 epidemiological data than with the inclusion of mobility pattern data.

### SHAP Analysis

**Figure 4.**
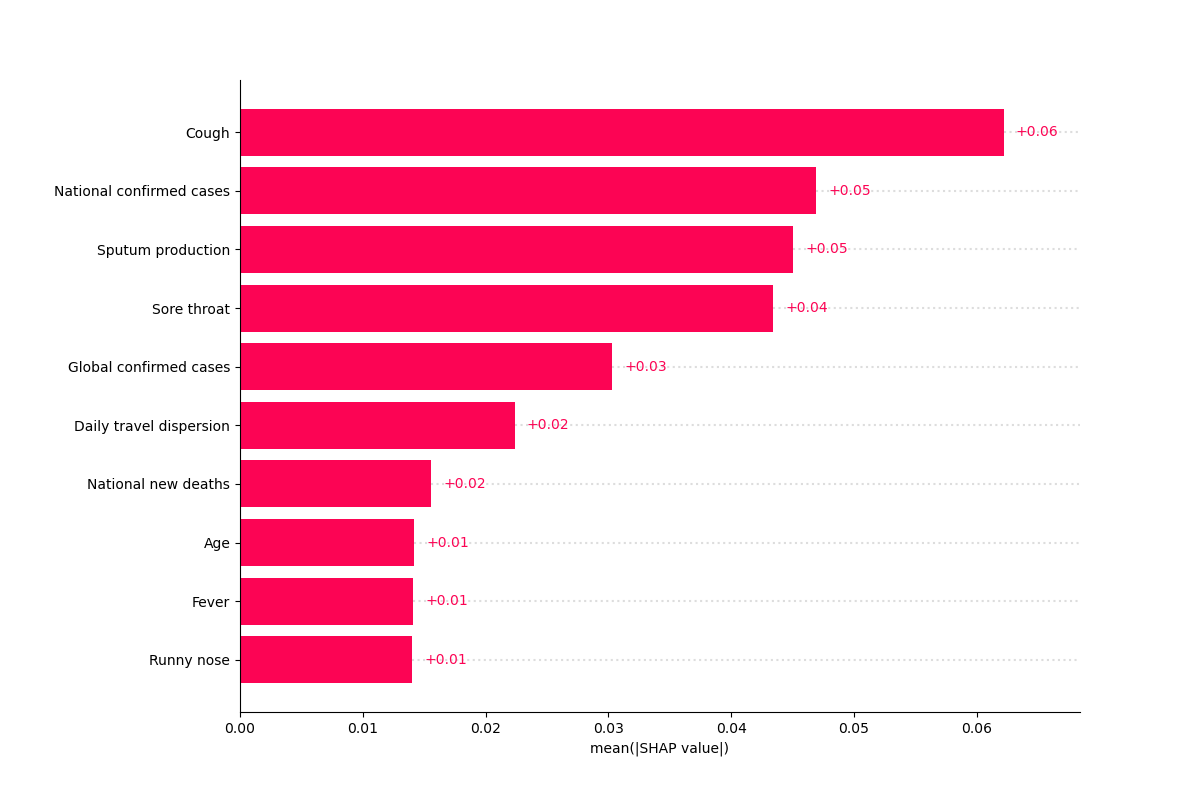
Top 10 important features, ranked by mean Shapley additive explanations values (Google AutoML model, “All” feature set) in the Study of Health Information for Next Epidemic dataset. National confirmed cases, global confirmed cases, and national new deaths are normalized values per million people. Daily travel dispersion refers to the travel patterns on the day prior to the test date. SHAP: Shapley additive explanations.

In Figure 4, cough emerged as the most influential predictor of COVID-19 positivity with a mean SHAP value of +0.06, followed by national confirmed cases (+0.05) and sputum production (+0.05). Sore throat ranked fourth (+0.04) and global confirmed cases fifth (+0.03). Mobility-related daily travel dispersion (+0.02) and national new deaths (+0.02) also contributed, whereas demographic variables such as age and fever showed only minor effects (+0.01 each). Respiratory symptoms, therefore, headed the importance ranking, with mobility and epidemiological indicators providing meaningful, but comparatively weaker, additional signals.

## Discussion

### Principal Findings

In this study, the accuracy of ML models for COVID-19 screening was significantly improved by the incorporation of external data, namely, mobility and epidemiological data, from the SHINE datasets. Our preliminary work with the Israeli dataset (Multimedia Appendix 1) provided initial support for this finding. The attribution of features in enhancing model performance was further reinforced by our SHAP analysis, which provided explainable evidence for the value of contextual data.

SHAP analysis showed that respiratory symptoms, such as cough, exerted the strongest influence on model predictions, whereas epidemiological and mobility variables ranked immediately after these symptoms and provided complementary but comparatively weaker predictive value. This highlights a key limitation of symptom-based screening: while symptoms such as fever or cough were significantly more prevalent in patients who tested positive for COVID-19, their overlap with other illnesses and the existence of thousands of asymptomatic positive cases reduced their diagnostic specificity. The synergy between contextual data (eg, exposure history, population movement patterns) and clinical features demonstrates that integrating diverse data sources—rather than relying on symptoms alone—can substantially improve screening accuracy and model reliability, particularly in asymptomatic or presymptomatic populations.

### Limitations

Our findings have certain limitations from the perspective of patient-generated health data. Although the volume of self-checked data collected was consistently maintained throughout the study period, we observed a concentrated accumulation of COVID-19 test submissions during the 2-month period between March 2022 and April 2022, in contrast to the even distribution of self-checked symptom submissions throughout the study duration. The higher number of tests recorded during this concentrated period can be attributed to increased awareness and concern regarding the use of the app and testing in the population. This is not particularly unusual, but it is important to consider that it may have contributed to model learning from our data during this specific period. Further, our data may have a selection bias due to government policies. For instance, during the data collection period, it was not possible to board return flights if individuals were infected with COVID-19 while overseas [20,39]. This suggests that in the future, when developing similar models for outbreaks of unprecedented epidemics such as the COVID-19 pandemic, incorporating the indicator of being a returning traveler from overseas may lead to a potential misjudgment of positivity probability.

According to our findings, incorporating mobility data has a positive impact on the performance of ML models. However, the metrics are subject to retrospective interpretation [40-42]. For instance, individuals who have already displayed severe initial symptoms of COVID-19 may find it more challenging to move actively. Furthermore, in our methodology, we focused on quantifying the extent of individuals’ activity levels rather than assessing the number of high-risk areas visited within that activity range.

### Conclusions

In conclusion, our study demonstrated the transformative potential of integrating mobility and epidemiological data into ML models for COVID-19 prediction. These external data sources not only improved the model accuracy but also highlighted the limitations of symptom-based predictions and the value of comprehensive data analysis. Our insights have practical implications for health care decision-making and public health interventions, paving the way for more informed responses to outbreaks.

## Supplementary material

10.2196/54956 Multimedia Appendix 1 Information about all variable lists, variable processing method, model hyperparameters, and preliminary experiment on Israeli dataset.

10.2196/54956 Checklist 1 CONSORT-AI checklist.

## Abbreviations

CONSORT-AI: Consolidated Standards of Reporting Trials–Artificial Intelligence
ML: machine learning
PCR: polymerase chain reaction
SHAP: Shapley additive explanations
SHINE: Study of Health Information for Next Epidemic
TabNet: tabular data network
