# Supplementary material for: Enhancing COVID-19 Screening Models With Epidemiological and Mobility Features: Machine-Learning Model Study
Source: JMIR AI. 2026 Mar 5;5:e54956. doi: 10.2196/54956 (PMC12978548; doi:10.2196/54956)
Supplement: Multimedia Appendix 1 [file ai-v5-e54956-s001.docx]

### **Detailed Table of Features Used in the Models**

The following tables present a detailed overview of all features used in our machine learning models for COVID-19 prediction. These features span multiple categories, including patient demographics, reported symptoms, test indications, mobility patterns derived from GPS data, and various epidemiological indicators at global, national, and regional levels. Each feature is categorized, named, and described, with its source provided, to offer a clear understanding of the data inputs to our models. This comprehensive set of features allows our models to capture a wide range of factors potentially influencing COVID-19 transmission and detection, from individual-level characteristics to broader population-level trends. We hope these tables serve as a reference and help other researchers gain a better understanding of the inputs to our models, thereby facilitating the reproducibility of our study.

#### Table 1: Features for the Preliminary Experiment with Israeli Dataset

| **Category** | **Source** | **Feature Name** | **Type** | **Description** |
| --- | --- | --- | --- | --- |
| Demographic | Israel | gender | Categorical | Patient's gender |
|  | Israel | age_60_above | Categorical | Binary feature indicating whether the patient is 60 years old or above (1) or below 60 years old |
| Symptoms | Israel | cough | Categorical | Presence or absence of cough |
|  | Israel | fever | Categorical | Presence or absence of fever |
|  | Israel | sore_throat | Categorical | Presence or absence of sore throat |
|  | Israel | shortness_of_breath | Categorical | Presence or absence of shortness of breath |
|  | Israel | sputum | Categorical | Presence or absence of sputum production |
|  | Israel | headache | Categorical | Presence or absence of headache |
| Test Indication | Israel | indication_other | Categorical | Whether the test was indicated for reasons other than travel or contact |
|  | Israel | indication_abroad | Categorical | Whether the test was indicated due to travel abroad |
|  | Israel | indication_contact | Categorical | Whether the test was indicated due to contact with a confirmed case |
| Global Epidemiological | OWID | global_confirmed_ratio | Numeric | Normalized confirmed COVID-19 cases to global population |
| National Epidemiological | OWID | confirmed_ratio | Numeric | Ratio of nationally confirmed COVID-19 cases to national population |
| Date-related | OWID | weekday | Categorical | Day of the week when the test was conducted |

#### Table 2: Features for the Main Experiment with SHINE Patient Dataset

| **Category** | **Source** | **Feature Name** | **Type** | **Description** |
| --- | --- | --- | --- | --- |
| Demographic | SHINE | gender | Categorical | Patient's gender |
|  | SHINE | age_ratio | Numeric | Patient's age, normalized to a ratio (e.g., 0-1 range) with max value 100 |
| Symptoms | SHINE | cough | Categorical | Presence or absence of cough |
|  | SHINE | fever | Categorical | Presence or absence of fever |
|  | SHINE | sore_throat | Categorical | Presence or absence of sore throat |
|  | SHINE | shortness_of_breath | Categorical | Presence or absence of shortness of breath |
|  | SHINE | sputum | Categorical | Presence or absence of sputum production |
|  | SHINE | headache | Categorical | Presence or absence of headache |
|  | SHINE | runny_nose | Categorical | Presence or absence of runny nose |
|  | SHINE | muscle_pain | Categorical | Presence or absence of muscle pain |
|  | SHINE | chills | Categorical | Presence or absence of chills |
|  | SHINE | loss_of_taste | Categorical | Presence or absence of taste loss |
|  | SHINE | loss_of_smell | Categorical | Presence or absence of smell loss |
|  | SHINE | chest_pain | Categorical | Presence or absence of chest pain |
|  | SHINE | fatigue | Categorical | Presence or absence of fatigue |
|  | SHINE | loss_appetite | Categorical | Presence or absence of appetite loss |
|  | SHINE | diarrhea | Categorical | Presence or absence of diarrhea |
| Test Indication | SHINE | indication_other | Categorical | Whether the test was indicated for reasons other than travel or contact |
|  | SHINE | indication_abroad | Categorical | Whether the test was indicated due to travel abroad |
|  | SHINE | indication_contact | Categorical | Whether the test was indicated due to contact with a confirmed case |
| Mobility (GPS) 21 features in total | SHINE | n_norm_gps-1 to n_norm_gps-7 | Numeric | Normalized number of unique GPS locations visited for each of 7 days prior to test |
|  | SHINE | loc_std_norm_gps-1 to loc_std_norm_gps-7 | Numeric | Normalized standard deviation of GPS locations for each of 7 days prior to test, indicating spread of movement |
|  | SHINE | path_sum_norm_gps-1 to path_sum_norm_gps-7 | Numeric | Normalized sum of path lengths from GPS data for each of 7 days prior to test, indicating total distance traveled |
| Global Epidemiological | OWID | global_confirmed_ratio | Numeric | Normalized confirmed COVID-19 cases to global population |
| National Epidemiological | OWID | confirmed_ratio | Numeric | Ratio of nationally confirmed COVID-19 cases to national population |
|  | OWID | total_cases_per_million_norm | Numeric | Normalized total COVID-19 cases per million population |
|  | OWID | new_cases_per_million_norm | Numeric | Normalized new daily COVID-19 cases per million population |
|  | OWID | new_cases_smoothed_per_million_norm | Numeric | Normalized 7-day average of new COVID-19 cases per million population |
|  | OWID | total_cases_per_million_for_6months_norm | Numeric | Normalized sum of daily total COVID-19 cases per million population over the previous 6 months |
|  | OWID | total_vaccinations_per_hundred_for_6months_norm | Numeric | Normalized sum of daily total COVID-19 vaccinations per hundred population over the previous 6 months |
|  | OWID | total_deaths_per_million_for_6months_norm | Numeric | Normalized sum of daily total COVID-19 deaths per million population over the previous 6 months |
|  | OWID | total_deaths_per_million_norm | Numeric | Normalized total COVID-19 deaths per million population |
|  | OWID | new_deaths_per_million_norm | Numeric | Normalized new daily COVID-19 deaths per million population |
|  | OWID | new_deaths_smoothed_per_million_norm | Numeric | Normalized 7-day average of new COVID-19 deaths per million population |
|  | OWID | icu_patients_per_million_norm | Numeric | Normalized number of COVID-19 patients in ICU per million population |
|  | OWID | hosp_patients_per_million_norm | Numeric | Normalized number of COVID-19 patients in hospital per million population |
|  | OWID | weekly_icu_admissions_per_million_norm | Numeric | Normalized weekly COVID-19 ICU admissions per million population |
|  | OWID | weekly_hosp_admissions_per_million_norm | Numeric | Normalized weekly COVID-19 hospital admissions per million population |
|  | OWID | total_tests_per_thousand_norm | Numeric | Normalized total COVID-19 tests conducted per thousand population |
|  | OWID | new_tests_per_thousand_norm | Numeric | Normalized new COVID-19 tests conducted per thousand population |
|  | OWID | new_tests_smoothed_per_thousand_norm | Numeric | Normalized 7-day average of new COVID-19 tests per thousand population |
|  | OWID | total_vaccinations_per_hundred_norm | Numeric | Normalized total COVID-19 vaccine doses administered per hundred population |
|  | OWID | people_vaccinated_per_hundred_norm | Numeric | Normalized number of people who received at least one vaccine dose per hundred population |
|  | OWID | people_fully_vaccinated_per_hundred_norm | Numeric | Normalized number of people fully vaccinated against COVID-19 per hundred population |
|  | OWID | total_boosters_per_hundred_norm | Numeric | Normalized total COVID-19 vaccine booster doses administered per hundred population |
|  | OWID | new_vaccinations_smoothed_per_million_norm | Numeric | Normalized 7-day average of new COVID-19 vaccine doses administered per million population |
|  | OWID | new_people_vaccinated_smoothed_per_hundred_norm | Numeric | Normalized 7-day average of newly vaccinated people per hundred population |
|  | OWID | hospital_beds_per_thousand_norm | Numeric | Normalized number of hospital beds per thousand population |
|  | OWID | excess_mortality_cumulative_per_million_norm | Numeric | Normalized cumulative excess mortality per million population since the start of the pandemic |
|  | OWID | reproduction_rate_norm | Numeric | Normalized estimated reproduction rate (R) of the virus |
|  | OWID | positive_rate_norm | Numeric | Normalized proportion of COVID-19 tests that are positive |
| Regional Epidemiological | SHINE | sigungu_confirmed_ratio | Numeric | Ratio of confirmed COVID-19 cases in the sigungu (district) to district population |
| Date-related | OWID | weekday | Categorical | Day of the week when the test was conducted |

For every feature in our dataset that has any missing values, we created a corresponding binary mask feature. This mask feature takes a value of 1 if the original feature's value is missing or masked, and 0 if the value is present and valid. This approach allows our models to explicitly learn patterns related to missing data, rather than relying on imputation methods that might introduce bias. By incorporating these mask features, we enable the models to distinguish between truly absent data and zero values, potentially capturing important patterns in data availability. The processing of GPS data, which required special handling due to its complex spatiotemporal nature, is detailed in the following section on mobility data processing.

**Mobility data pre-processing**

Mobility data

Mobility data were obtained by processing the GPS tracking data collected from individual users via the SHINE app. Instead of utilizing raw GPS positions, we computed secondary metrics such as the radius of movement and total distance traveled. Subsequently, these metrics were mapped to the original episodes based on their associated dates.

In this study, the input data were preprocessed as follows: (1) grouping by day; (2) generating location-related secondary features daily; and (3) regularizing each secondary feature based on its individual characteristics. The location data were presented as a three-dimensional vector. The date of each geolocation was denoted as d and the index distinguishing the geolocation within that date as i. With these notations, the geolocation is depicted as follows:

$$g_{d,i} = \left( \phi_{d,i}, \lambda_{d,i}, t_{d,i} \right)$$

where ϕ is the latitude of the GPS signal, λ is the longitude, and t is the timestamp.

A set G(d) comprising daily collections of location data g was then constructed and used to produce a vector v, which was eventually utilized as an input to the model. This was achieved using a preprocessing function f(G) in various forms. We conceptualized v as an ordered pair stemming from the preprocessing functions applied to the daily set G(d) and secondary features x, which were normalized across the entire dataset. Let f_j be the jth pre-processing function.

$$G(d) = (g_{d, 1} , g_{d, 2} , g_{d, 3} , ... )$$

$$v_{d,j} = norm(f_{j}(G(d)))$$

The resulting vector was utilized as input data by integrating information such as a user's symptoms from various preceding dates (e.g., 1, 2, and 3 days ago) based on the date of input.

$$v =(v_{-1}, v_{-2}, v_{-3}, ... v_{d})$$

In this study, we employed three functions to generate features; hence, in the experiments, j is at most three. These functions were (1) number of GPS signals captured daily, (2) daily moving distances derived from recorded GPS signals, and (3) the dispersion of movement calculated from the captured GPS signals. The preprocessing functions and normalization methods used to generate each feature are detailed below.

Preprocessing functions

Number of geographical measurements

The number of geolocation measurements on day d, n_loc , was defined as follows:

$$f_{1} = N(G(d))$$

Traveling distance per day

The total distance traveled using location information was represented as the sum of each distance when the location data for a particular day were linked chronologically. The formula used was as follows:

$$f_{2} = \sum_{i=1}^{n-1} \left| g_{d,i+1} - g_{d,i} \right|$$

The distance between the two GPS signals, G1 and G2, is computed on the sphere using the Haversine formula:

$$a = sin^{2}\left( \Delta\phi/2 \right) +cos \phi_{1}*cos \phi_{2} * sin^{2}\left( \Delta\lambda/2 \right)$$

$$c = 2 * atan2\left( \sqrt{a}+\sqrt{\left( 1-a \right)} \right)$$

$$\left| g_{1}-g_{2} \right|=\sqrt{R * c}$$

where l1 represents latitude; l2, longitude; and R, the earth’s radius.

Measurement of dispersion

The average distance from a central point is evaluated according to the metric loc_std (a measure of dispersion) determined using the following formula:

$$f_{3} =\sqrt{Var(\phi_{d}) + Var(\lambda_{d})}$$

where Var(x) is the variance of x values

$$Var(x) =\frac{1}{n}\sum_{i=1}^{n} (x_{i} - x_{c})^{2}$$

where xc is the mean value of the latitude or longitude of the GPS record

Normalization method

Most features had long-tailed distributions with extreme values, making it necessary to use appropriate normalization methods. In this study, experiments were performed using two methods: quantile normalization and log normalization.

Log normalization

Given the large number of extreme values, each measurement was logarithmized. It was then normalized using the mean and standard deviation, such that the mean was 0 and the standard deviation was 1.

$$y_{i} = log\left( x_{i}+\rho\right)$$

$$log\_norm(x_{i}) = \frac{y_{i}- Mean(y)}{SD(y)}$$

where ρ is the smoothing factor. In this experiment, we used ρ=1.

Quantile normalization

The Quantile Transformer from Scikit-learn version 1.1.2 was used.

$$qt\_norm(x_{i}) = QuantileTransform(x_{i})$$

There were no significant differences in the results with experiments under these two normalization methods. However, the results obtained using quantile normalization were included in the final presentation.

**Training and model hyperparameters**

The input data format for training and validation was formed as a 1-D vector by concatenating all the features listed below. For the training data, the input vector format was V x B (batch size), where V represents the total number of features and B represents the batch size.

1. Symptom and Patient Features

We created an input vector from the symptoms reported by each user and included basic patient information such as gender, age, and reason for testing. Except for age, all other features were represented as a multi-labeled binary vector. For the SHINE dataset, the symptom and patient features formed a 26-dimensional vector, while for the Israeli dataset, they formed a 10-dimensional vector due to the difference in the number of symptoms reported.

1. Epidemiological Features

We constructed three epidemiological indices using OWID data and regional government announcements:

- 'global_confirmed_ratio'
- 'confirmed_ratio'
- 'sigungu_confirmed_ratio'
- 'total_cases_per_million_for_6months_norm'
- 'total_vaccinations_per_hundred_for_6months_norm'
- 'total_deaths_per_million_for_6months_norm'

Additionally, we directly used the following features from the OWID data from the day before the assessment date:

- reproduction_rate
- positive_rate
- weekly_icu_admissions_per_million
- total_vaccinations_per_hundred
- total_tests_per_thousand
- total_deaths_per_million
- new_vaccinations_smoothed_per_million
- new_people_vaccinated_smoothed_per_hundred
- total_cases_per_million
- new_tests_smoothed_per_thousand
- hosp_patients_per_million
- icu_patients_per_million
- new_cases_per_million
- weekly_hosp_admissions_per_million
- new_tests_per_thousand
- excess_mortality_cumulative_per_million
- new_deaths_smoothed_per_million
- people_fully_vaccinated_per_hundred
- people_vaccinated_per_hundred
- new_cases_smoothed_per_million
- total_boosters_per_hundred
- hospital_beds_per_thousand
- new_deaths_per_million

1. Mobility Data Features

As mentioned in the "Mobility Data Pre-processing" section, each day of the mobility data was transformed into a 3-dimensional vector. In this training, we used 7 days of mobility features after pre-processing, resulting in a 21-dimensional vector in total. We concatenated this vector with the patient feature vector and epidemiological feature vector.

1. Mask for Missing Values

To distinguish missing values during training, we added a 'mask' column for each feature that included missing or erroneous values. By adding this feature, the machine learning model can differentiate between missing/null values and zero values.

Including all the above features, the dimension of the largest vector in our experiment was 68. You can find the list of all features in the source code here: https://github.com/mobiledoctorDev/SHINE_patient_is_not_all_you_need/blob/main/train_scripts/model/features.py

In our experiments, model performance varied depending on the model characteristics. To mitigate this, we assessed the performance across six distinct models: logistic regression, XGBoost, LGBM, TabNet, mTAN, and Google AutoML.

The logistic regression model can be conceptualized as the weighted sum of variable differences along with a sigmoid function. Its strength lies in its simplicity, which allows clearer insights into the relationships between the variables and outcomes. We employed the logistic regression model from the Python scikit-learn package (version 1.1.1) with the following default parameters: L2 penalty, dual formulation, liblinear solver, tolerance of 1e-4, and regularization strength of 1.0.

We also utilized two non-neural network-based machine learning models, namely, XGBoost and LightGBM. Python libraries lightgbm (version 3.1.1) and xgboost (version 1.7.2) were used. Within the LightGBM library, we used the LGBMClassifier with default parameters: boosting_type='gbdt,’ num_leaves=31, max_depth set to "no limit,” learning_rate=0.1, and n_estimators=100. For XGBoost, the library's XGBRegressor was employed with the following parameters: the number of estimators (M) was set to 10, a linear learner objective function was used, and other parameters were set to default settings.

The TabNet model, a deep-learning framework designed for tabular data, was also utilized. TabNet employs a unique structure named attentive transformer and enhances performance through unsupervised learning. We used the tab_model.TabNetClassifier from the Python Pytorch _tabnet library (version 4.0). All parameters were set to the library's default values (n_d=8, n_a=8, n_steps=3, gamma=1.3, and lr=2e-2).

Finally, we implemented the Google Cloud AutoML model. This model utilizes features from Vertex AI. The same training/validation/test dataset structure from the other tabular training models were applied to this model. The AutoML model was trained using the "node time 2" option, and the AUROC on the evaluation page served as the performance metric. Consistent weights for the training data and features were maintained.

It is crucial to note that although there was an imbalance between positive and negative episodes, we refrained from employing oversampling or downsampling techniques. To minimize the potential confounding risks, episodes from the same individual were grouped into the test or the training set.

### **Preliminary Analysis with the Israeli Dataset**

Background

Prior to our main analysis with the SHINE dataset, we conducted a preliminary experiment using a public dataset from Israel to assess whether incorporating epidemiological features could enhance COVID-19 prediction performance. This preliminary work provided initial support for our hypothesis that contextual information beyond patient symptoms could improve screening accuracy.

Data Source

The Israeli dataset consisted of COVID-19 test results from Israel and encompassed valuable information, such as symptoms, age, sex, and reasons for testing. This dataset was publicly released by the Israeli Ministry of Health and can be accessed via https://data.gov.il/dataset/covid-19. Data collected until June 30, 2023, were utilized for this study.

To test the hypothesis that integrating prevalence information could enhance individual diagnosis, we conducted a preliminary experiment based on the public data from the Israel Ministry of Health. This dataset was supplemented with aggregated data from health authorities, particularly on the number of confirmed COVID-19 cases. We also incorporated relevant contextual information from Our World in Data (OWID), including global and national surveillance data.

Characteristics of the Israeli Dataset

Table S1. Comparison of COVID-19 Testing Results Between the Israeli and the SHINE Datasets.

| Category | Sub-category | Israeli dataset (N = 6,791,352) |
| --- | --- | --- |
| **Age, years** | <60 | 5,887,828 (86·69%) |
|  | ≥60 | 903,524(13·31%) |
| **Sex** | Female | 3,506,667 (51·63%) |
|  | Male | 3,284,685 (48·27%) |
| **Test indication^1^** | Others | 6,150,448 (90·56%) |
|  | Contact^2^ | 599,655 (8·82%) |
|  | Abroad^3^ | 41,249 (0·62%) |
| **Test result** | Negative | 5,916,638 (87·12%) |
|  | positive | 874,714 (12·88%) |

^1^ Reasons prompting COVID-19 testing.

^2^ Close contact with a confirmed COVID-19 case.

^3^ Recent return from international travel.

Methods

To test our hypothesis with the Israeli dataset, we incorporated additional epidemiological features, specifically global confirmed cases and national (Israeli) confirmed cases, as additional features in our predictive models. These epidemiological features were incorporated alongside standard patient symptom data in a logistic regression model.

In our preliminary experiment, we aimed to demonstrate potential improvements in the predictive performance of the model by incorporating readily available COVID-19 epidemiological data from the Israeli dataset. Global confirmed cases and national (Israeli) confirmed cases were incorporated as additional features of the models.

Results

We examined the influence of epidemiological features, the global confirmed cases and the national confirmed cases, on the prediction of individual COVID-19 infection statuses using the Israeli dataset. Incorporation of the epidemiological features in the logistic regression model significantly improved the model performance. The inclusion of epidemiological features in the model led to a substantial increase in the mean AUC, with values rising from 0.6704 (SD: 0.003) to 0.8144 (SD: 0.002), representing an improvement of 0.144.

Notably, epidemiological factors, including global and national confirmed cases, exhibited similar levels of significance in comparison to primary features such as symptoms like fever and cough, as depicted in Figure S1.


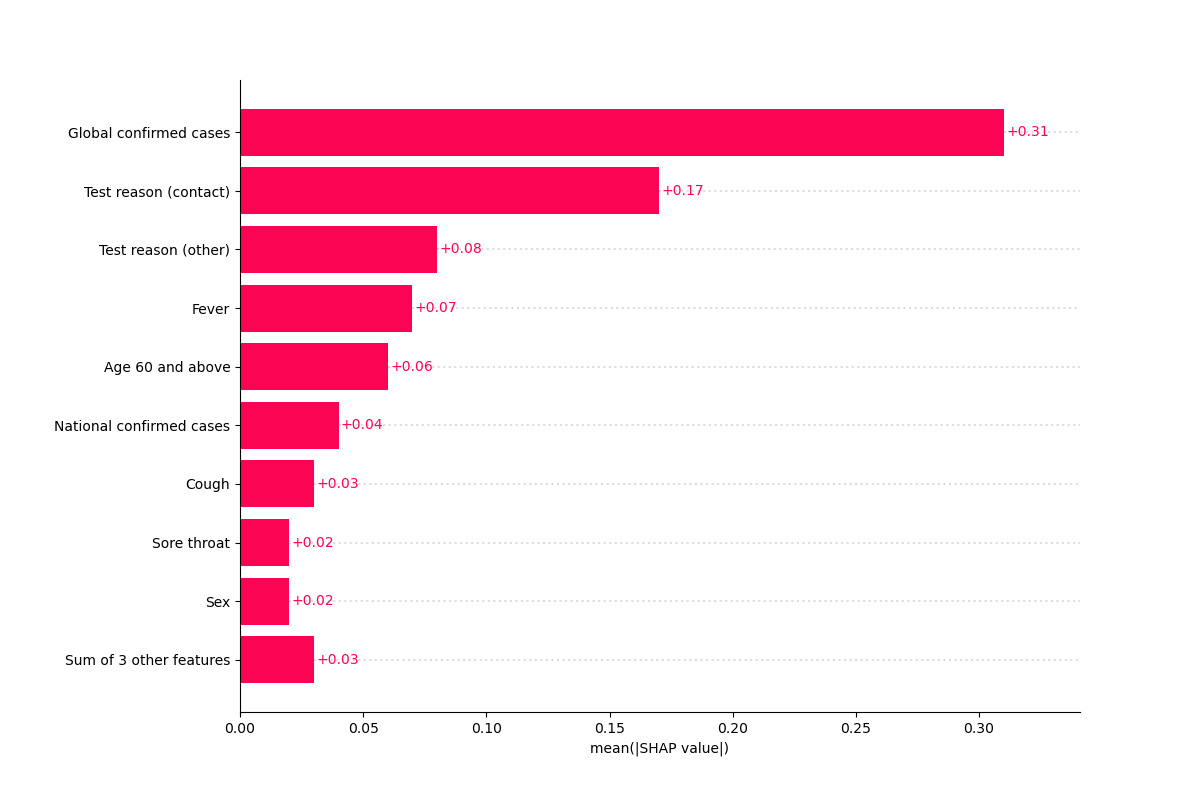


[Figure S1: Feature importance analysis from the Israeli dataset showing the relative importance of symptoms and epidemiological features in the logistic regression model.]

Discussion

Our preliminary analysis with the Israeli dataset provided initial evidence that incorporating epidemiological data alongside patient symptom data could significantly improve COVID-19 prediction performance. The substantial increase in AUC (from 0.6704 to 0.8144) demonstrated the value of including contextual epidemic information in diagnostic models.

This finding was particularly notable because epidemiological factors (global and national confirmed cases) showed similar importance levels to primary symptom features, suggesting that population-level context is as crucial for accurate prediction as individual symptoms. This preliminary work laid the foundation for our more comprehensive analysis with the SHINE dataset, which incorporated additional mobility data and more granular epidemiological information.
