# Supplementary material for: Enhancing COVID-19 Screening Models With Epidemiological and Mobility Features: Machine-Learning Model Study
Source: JMIR AI. 2026 Mar 5;5:e54956. doi: 10.2196/54956 (PMC12978548; doi:10.2196/54956)
Supplement: Checklist 1 [file ai-v5-e54956-s002.pdf]

Consolidated reporting guidelines for prognostic and diagnostic machine learning modeling studies

Multimedia Appendix 1: Author Checklist

The following is the reporting checklist. A response should indicate whether the particular item is documented in the study. If the response to an item is Y then the location in the article should be provided (e.g., section number), and if the response is N or NA then some reasoning should be provided.

#	Item	Y	N	NA	Location / Reasoning
**Study Details**					
1.1	*The medical/clinical task of interest*	X			Introduction
1.2	*The research question*	X			Introduction, Methods/Study design
1.3	*Current medical/clinical practice*	X			Introduction
1.4	*The known predictors and confounders to what is being predicted / diagnosed*			X	N/A
1.5	*The overall study design*	X			Methods/Study design
1.6	*The medical institutional setting(s)*	X			Methods/SHINE dataset
1.7	*The target patient population*	X			Results/SHINE dataset characteristics
1.8	*The intended use of the ML model*	X			Methods/Study design
1.9	*Existing model performance benchmarks for this task*			X	No existing model for this task
1.10	*Ethical and other regulatory approvals obtained*	X			Methods/SHINE dataset
**The Data**					
2.1	*Inclusion / exclusion criteria for the patient cohort*	X			Methods/SHINE dataset
2.2	*Methods of data collection*	X			Methods/SHINE dataset
2.3	*Bias introduced due to the method of data collection used*	X			Discussion/Limitations
2.4	*Data characteristics*	X			Results/SHINE dataset characteristics
2.5	*Methods of data transformations and preprocessing applied*	X			Methods/SHINE dataset, supplementary
2.6	*Known quality issues with the data*	X			Discussion/Limitation
2.7	*Sample size calculation*	X			Results/SHINE dataset characteristics

2.8	*Data Availability*	X			Data sharing and availability
**Methodology**					
3.1	*Strategies for handling missing data*	X			Supplementary material
3.2	*Strategies for addressing class imbalance*		X		
3.3	*Strategies for reducing dimensionality of data*		X		
3.4	*Strategies for handling outliers*		X		
3.5	*Strategies for data augmentation*		X		
3.6	*Strategies for model pre-training*		X		
3.7	*The rationale for selecting the machine learning algorithm*	X			Methods/Models and training/Model selection
3.8	*The method of evaluating model performance during training*	X			Methods/Models and training/Performance evaluation
3.9	*The method used for hyperparameter tuning*		X		We do not apply any hyperparameter tuning, as our focus is not present best performance
3.10	*Model's output adjustments*		X		
**Evaluation**					
4.1	*Performance metrics used to evaluate the model*	X			Methods/Models and training/Performance evaluation
4.2	*The cost or consequence of errors*		X		
4.3	*The results of internal validation*	X			Results/ Impact of mobility and epidemiological feature addition on COVID-19 prediction in the SHINE dataset
4.4	*The final model hyperparameters*	X			Supplementary 1
4.5	*Model evaluation on an external dataset*			X	No available external data exists

4.6	*Characteristics relevant for detecting data shift and drift*			X	This is not a product level development
**Explainability and Transparency**					
5.1	*The most important features and how they relate to the outcome(s)*	X			Results/ Impact of mobility and epidemiological feature addition on COVID-19 prediction in the SHINE dataset
5.2	*Plausibility of model outputs*		X		This is not a product level development
5.3	*Interpretation of model's results by an end-user*		X		This is not a product level development
